# Endophytic and epiphytic microbes as “sources” of bioactive agents

**DOI:** 10.3389/fchem.2015.00034

**Published:** 2015-05-22

**Authors:** David J. Newman, Gordon M. Cragg

**Affiliations:** ^1^Retired, Wayne, PA, USA; ^2^Retired, Bethesda, MD, USA

**Keywords:** endophyte, epiphyte, natural product sources, ultured microbes, novel sources

## Abstract

Beginning with the report by Stierle and Strobel in 1993 on taxol^(R)^ production by an endophytic fungus (Stierle et al., 1993), it is possible that a number of the agents now used as leads to treatments of diseases in man, are not produced by the plant or invertebrate host from which they were first isolated and identified. They are probably the product of a microbe in, on or around the macroorganism. At times there is an intricate “dance” between a precursor produced by a microbe, and interactions within the macroorganism, or in certain cases, a fungus, that ends up with the production of a novel agent that has potential as a treatment for a human disease. This report will give examples from insects, plants, and marine invertebrates.

## Introduction

Due to the differences in timing of reports in the literature, we have attempted to identify when the first report of endo- or epiphytic microbes being involved in the production of a particular compound, or class of compounds, isolated from a host organism was reported. As mentioned in the abstract, we will cover, albeit only superficially in some cases, developments from a descriptive aspect, but essential citations will be given so that interested readers can investigate further. In addition to the three sources given in the abstract, we will also comment on some very interesting, relatively recent relationships between fungi and bacteria, a relationship that is not usually recognized.

## Marine sourced materials

**Figure 1:**
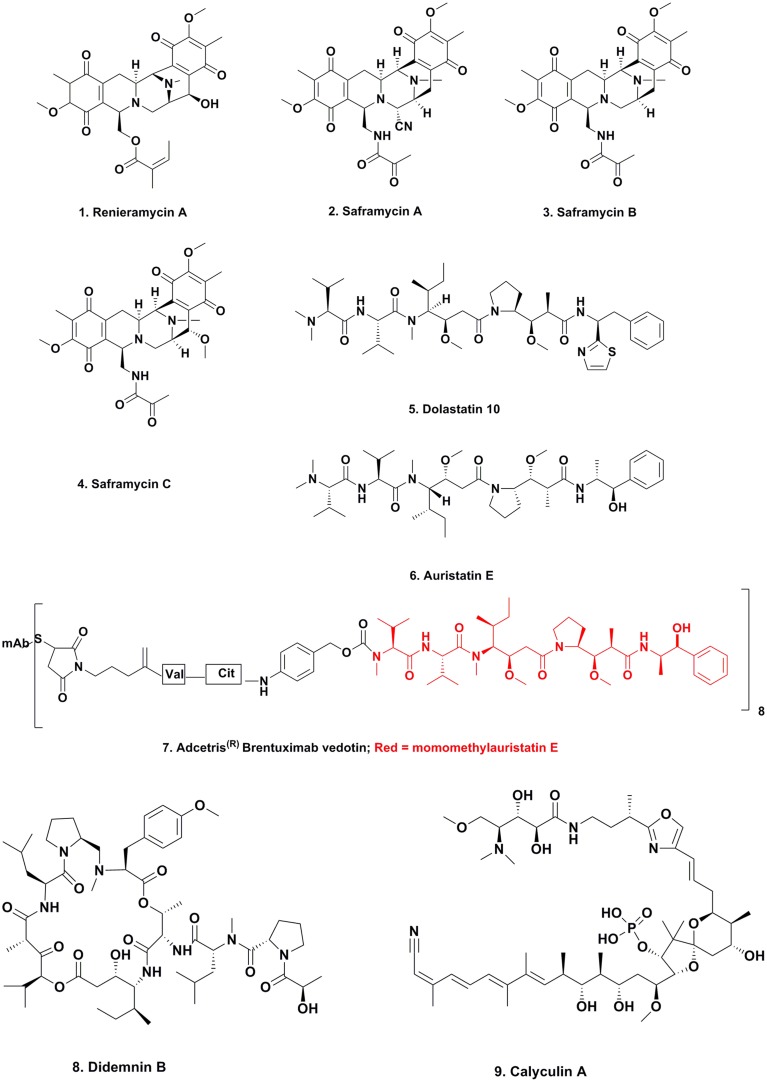
**Compounds from Marine-sourced Microbes**.

In the early 1980s, Frincke and Faulkner (1982) from the Scripps Institution of Oceanography in California investigating the compounds produced (better terms today might be “found in” or “isolated from”) by sponges in the Eastern Pacific off the West coast of California, isolated, and purified the compound known as renieramycin A (Figure 1; **1**). Inspection of the structure of this molecule showed that the base structure closely resembled a series of known antitumor agents that had been isolated from fermentation of a terrestrial microbe, the saframycins A–C (Figure 1; **2–4**). These compounds had been reported (Arai et al., 1977) from *Streptomyces lavendulae* initially as antibiotics, and later as having antitumor activity (Arai et al., 1980). Faulkner was not able to determine the antitumor activity of his isolate due to the very small amount of material isolated. Twenty years later, the Fusetani group in Tokyo (Nakao et al., 2004) reported the same material from an entirely different sponge, a *Neopetrosia* species using an antileishmanial assay rather than an antitumor assay; thus demonstrating that the same molecule may well have quite different activities dependent upon the bioassay used for following the isolation. It may be relevent at this point to make the point that most of the marine-derived materials reported in the literature were identified by bioactivity driven isolation techniques.

This series of discoveries could be considered the beginnings of a tsunami of reports over the last 30 plus years, that now have led to the possibility that the majority of compounds isolated from multicellular marine invertebrates involve production by a microorganism. We have used the term multicellular to differentiate from single celled organisms, though even that definition might be incorrect as knowledge evolves. The production may have, but equally may not have an interaction with the nominal “host producer.”

We will now give some specific examples of what we have just described; these will to some extent be in chronological order by discovery of the original compound, but the “proof” (direct or in some cases circumstantial) has occurred at differing time points from the original report(s).

## Dolastatins

This collection of linear and cyclic peptides with very unusual amino acids in their structures, were first described by the Pettit group at Arizona State University working in conjunction with the National Cancer Institute, using the NCI's bioactive assays initially, and were shown to have potent antitumor activities. Due to the very limited abundance of the nudibranch from which they were first isolated, once the initial structures were determined, the molecules had to be synthesized chemically in order to advance them into preclinical development, and then into clinical trials as an antitumor agent in the case of dolastatin 10 (Figure 1; **5**). The full details of the initial discoveries and synthetic methodologies were well described by Flahive and Srirangam (2012).

In the late 1990s to early 2000s, the Hawaiian group led by Moore reported that the probable producer of these molecules was a cyanobacterium on which the nudibranch grazed. Thus, dolastatins 3, 10, and 12 were reported from cyanobacteria (Luesch et al., 2002) and although not formally reported in the literature, Dr. Valerie Paul (then at the University of Guam Marine Station) observed *D. auricularia* (the nudibranch from which the dolastatins were originally isolated) grazing upon cyanobacteria containing dolastatins, She subsequently isolated dolastatins from both the nudibranch and the cyanophyte (Paul, personal communication).

Although none of the naturally occurring dolastatins successfully transitioned from discovery to a clinically approved drug, an analog that was based upon the dolastatin 10 structure has become an approved antitumor drug. The modified dolastatin now known as vedotin, based on auristatin E (Figure 1; **6**), was used as a warhead on a monoclonal antibody directed against Hodgkins lymphoma. This combination, known as Adcetris^(R)^ (Figure 1; **7**) was approved in 2011 by the US FDA, but would never have been synthesized in the absence of the knowledge of the dolastatin structures. As of early 2014 there were 21 variations (different combinations of auristatin E or F and different MAbs/linkers) in clinical trials or close to entering them (Newman and Cragg, 2014). Currently (March 2015) there are nine combinations of monoclonal antibodies linked to auristatin E in Phase I to Phase III clinical trials, and two using auristatin F in Phase I trials against cancer targets. The “drop-out” of molecules at the Phase I level is very frequent, so the difference in numbers is not unusual.

## Didemnins

The first marine-derived agent to go into clinical trials for cancer was the cyclic depsipeptide didemnin B (Figure 1; **8**). This was one of a number of very similar compounds reported by the Rinehart group at the University of Illinois in the early 1980s from the tunicate *Trididemnum solidum*. As with the dolastatins, a total synthesis was necessary in order to obtain enough material for preclinical and clinical trials, and this was reported in 1987 (Rinehart et al., 1987).

The compound progressed through to Phase II clinical trials but did not proceed beyond this level due to a combination of lack of activity and toxicity. Full details of the synthetic methods and the clinical development was published by Lee et al. (2012).

What was a major discovery as to the source came from two papers, one from Japan published in 2011 (Tsukimoto et al., 2011) demonstrating that a free-living microbe from Japanese waters produced didemnin B, and the other reported by a Chinese-Saudi-USA consortium giving the full genomic sequence of the didemnin gene cluster from a microbe collected in the Red Sea (Xu et al., 2012). This later paper demonstrated the temporal production of the didemnins via previously suggested intermediates in “real time” by using mass spectrometric techniques on the growing microbe (Xu et al., 2012). The free-living microbes in both cases were from the unusual genus, *Tistrella* with *T. mobilis* in the first report and *T. bauzanensis* and *T. mobilis* in the second. Thus, there is no doubt that these are the source of these depsipeptides.

A very close chemical relative, aplidine was isolated from the same tunicate differing only by two hydrogen atoms on the side chain, with a pyruvyl instead of a lactyl group as in didemnin B. This compound was later found in the Mediterranian tunicate *Aplidium albus* by PharmaMar scientists, and is currently in multiple clinical trials from Phase II to Phase III with PharmaMar. An MAA (equivalent to the US NDA) filing is due in 2015.

## Ecteinascidin 743

In the 1969/1970 time frame, Sigel and colleagues reported on the antitumor activity of an ethanolic extract of the tunicate *Ecteinascidia turbinata* [published in book form in 1970 (Sigel et al., 1970)]. The active compounds, all with the base skeleton of the napthyridinomycin alkaloids exemplified by the saframycins (tetrahydroisoquinoline alkaloids), were then isolated from the same Caribbean tunicate *E. turbinata* 17 years later as a complex of similar molecules. The first formal report was by Holt in his PhD thesis in 1986 (Holt, 1986). This was followed in 1990 by two simultaneous reports, one from the Rinehart group at the University of Illinois (Rinehart et al., 1990) and the other from the Wright group at Harbor Branch Oceanographic Institution (Wright et al., 1990). The molecules were licensed to the Spanish company PharmaMar for preclinical and clinical development, being approved in the EU in 2007 as Yondelis^(R)^ for the treatment of sarcoma. In November, 2014 an NDA was filed in the USA by Janssen (who licensed the molecule) for the same indication. The full story of the production by aquaculture and then semisynthesis was reported by Cuevas and Francesch (2009) and Cuevas et al. (2012), and should be consulted for further information.

Although the production of the molecule for clinical use was via semisynthesis from cyanosafracin (a cyano derivative of a microbial metabolite), there were suggestions that an as yet uncultured bacterium, *Candidatus Endoecteinacidia frumentenis* (AY054370), was involved in the production of these molecules. This organism was found in ecteinascidin 743 producing *E. turbinata* collected in both the Caribbean and the Mediterranean (Moss et al., 2003; Perez-Matos et al., 2007). These reports, coupled to the suggestions by Piel (2006) as to how to utilize bacterial symbionts from invertebrates, led to the confirmation of these suggestions by Rath et al. (2011).

By using the known gene clusters of the saframycin (Li et al., 2008) and safracin (Velasco et al., 2005) metabolites as markers, the “contig” encoding the NRPS biosynthetic enzymes involved in trabectedin production was identified as well as the producing organism. This was the γ-proteobacterium known as *Candidatus Endoecteinascidia frumentensis* (AY054370), previously suggested as the actual producer even though not yet cultured. An example of what can now be done using advanced genomic techniques.

### Candidatus entotheonella

#### Metabolite production in the sponge theonella swinhoei

The work reported in the journal Nature by the Piel group early in 2014 on the production of metabolites from the yellow or “Y” biotype of this sponge, effectively laid to rest circumstantial arguments about sponge metabolites being derived from microbes in the sponge (Wilson et al., 2014). In a tour-de-force, this group isolated two phylotypes of the candidate genus *Entotheonella* with genomes greater than nine megabases and multiple distinct biosynthetic gene clusters from this sponge, via cell disruption and FACS sorting into reaction wells with a single cell per well. From genomic studies, 31 of the reported 32 polyketide metabolites (most of which have reported bioactivity) previously isolated from this sponge variant were attributed to a single phylotype. These as yet uncultured bacteria are widely distributed in sponges and belong to an environmental taxon proposed as the candidate phylum *Tectomicrobia*.

#### Calyculin production in *Discodermia calyx*

Almost simultaneously with the *Theonella swinhoei* results, a similar series of experiments, but looking at the production of the well-known phosphatase inhibitor, calyculin (Figure 1; **9**) isolated from the sponge *Discodermia calyx*, demonstrated that the molecule was in fact produced by the symbiotic bacterium, *Candidatus Entotheonella* sp. A (Wakimoto et al., 2014).

The potential for use of these gene clusters in the production of previously known and unknown metabolites is discussed in the recent papers by Helfrich et al. (2014) and Guo et al. (2015) which should be consulted for examples. These are not the only papers dealing with this subject but they are amongst the most recent.

#### Plants and endophytes/epiphytes

From our perspective, the situation with plants, and whether or not microbes have anything to do with the metabolites found from studying compounds isolated from plant materials, is now roughly at the same stage of “proof” as the situation which existed 2 or 3 years after the initial discovery by Faulkner's group of the renieramycins (*vide infra*). This applies to compounds isolated from plants either by using bioactivity-driven isolation, or by what used to be known as “grind and find/phytochemical investigations,” where compounds were isolated and then sometimes the purified chemical entities would be investigated pharmacologically.

The major difference is that the discovery of renieramycin closely followed the beginning of the systematic discovery of metabolites in organisms from the marine environment, whereas roughly two centuries had elapsed between pure compound discovery from terrestrial plants, dating approximately from Seturner's identification of purified morphine in 1817, and the discovery of potential microbial involvement in plant metabolite production. We should note that there are conflicting reports as to the dates recorded in the literature for the isolation of morphine, which range from 1803 to 1817, but the full chronology showing that the initial 1803-04 report was not the isolation of an alkaloid (basic) but rather an acidic compound (possibly meconic acid) has been given by Newman and Cragg in 2010, and should be consulted for the full story (Newman and Cragg, 2010).

In 2003, Strobel suggested that every one of the approximately 350,000 species of vascular plants on Earth serves as a host for at least one endophytic microbe, organisms (often fungal in nature) that live within the tissues of the plant but do not cause any deleterious effect on the plant host. This suggestion was possibly due to his initial work on the microbial production of taxol^(R)^ by an endophytic fungus originally isolated from the inner tissues of the taxol-producing *Taxus brevifolia* tree and reported in 1993 (Stierle et al., 1993).

Does this comment mean that plants do not produce secondary metabolites but that microbes are involved in every facet of production? No, this is not our contention at this state of knowledge.

What we will show in this section is that in the case of some well-known compounds with a variety of pharmacological activities, the actual producers are in some cases a microbe (often fungal in origin), and in other cases, microbes are involved but variable results are obtained on fermentation of the microbe at this stage of knowledge.

In some cases, there are reports of isolated microbes not known to be involved with a plant producing what were thought to be “plant-only” compounds such as chalcones. An excellent example would be the work reported by Moore et al. in 2002 demonstrating the presence of Type III PKS systems in the marine bacterium *Streptomyces maritimus* (Moore et al., 2002).

#### Taxol^**(R)**^ from endophytes

As mentioned above, the report on the potential of isolated fungi to produce secondary metabolites that were in low quantity in the host plant, caused a substantial number of natural product chemists and biologists to start investigating, not only the production of taxol^(R)^, but also to look at other pharmacologically interesting molecules which will be considered in subsequent sections.

In the case of taxol^(R)^ there have been many publications over the last 20 years where investigators have demonstrated that low levels of taxol^(R)^ could be obtained from many endophytic fungi isolated, not just from *Taxus* species but from a multiplicity of plants, even including hazelnut producing plants, first reported in 2000 (Service, 2000).

In the case of the hazelnut, much more information plus transcriptome analyses were published by Ma et al. (2013), demonstrating the genes necessary for taxol^(R)^ biosynthesis. Recently, Yang et al. identified paclitaxel production in an endophyte, *Penicillium aurantiogriseum* from hazel and identified the gene clusters involved, demonstrating evolution of the biosynthetic machinery in this *Penicillium* species independent of the plant host (Yang et al., 2014). In this case, there is little doubt that the fungus produces the compound.

Although one paper was recently published that claimed not to be able to identify any taxane biosynthesis in three fungi (including the original isolate from Strobel, though obtained from a repository, not the original investigators) and two that they isolated themselves from *Taxus* species (Heinig et al., 2013), many other investigators have been able to obtain genetic information including the full biosynthetic pathway from endophytic fungi.

The following recent papers should be consulted for the results demonstrating production of taxol^(R)^ by a variety of endophytic fungi including identification of the relevant genetic machinery in the fungi investigated (Zaiyou et al., 2013; Kusari et al., 2014a,b). These papers demonstrate the potential, and the Soliman and Raizada paper in 2013 is of significant interest because it points out that the experiments utilized in all previous work relied upon axenic culture methods, whereas in the plant there would be significant interaction/competition between different microbes. They demonstrated increased yields when competitive fungi and other agents were introduced into the cultures, a phenomenon known to “induce” expression of cryptic gene clusters (Soliman and Raizada, 2013). One excellent example of this type of response is the report where suspension cells of *Taxus chinensis* var *mairei* were co-cultured in a bioreactor with its endophytic microbe, *Fusarium mairei* with a doubling of the yield of taxol^(R)^ (Li et al., 2009).

Thus, we consider that there is sufficient evidence to implicate fungal endophytes in the production of taxol^(R)^ in plants but the fungi so far investigated, except in the case of *Penicillium* and hazel, may not be the only “player(s)” in the system, since as mentioned above the genes required for taxol^(R)^ biosynthesis may well require activation of cryptic clusters in the interacting microbe(s). Many examples, though not from this system, have been published (Bertrand et al., 2014; Whitt et al., 2014), and recently Kusari et al., published a paper that covered interactions across a variety of kingdoms and phyla relevant to this thesis (Kusari et al., 2013).

One consistent comment made by reviewers/authors arguing against fungal/microbial involvement in taxol^(R)^ production in plants is that a major source of this compound for commercial use is plant tissue culture. However, to the authors' knowledge, there are no axenic plant tissue culture processes for any “plant-derived metabolite.” Thus, until an axenic (not surface sterilized or aseptic) plant tissue culture process that produces a metabolite is proven, microbes can still be involved.

#### Non-taxanes

In a recent short review paper, investigators in Proksch's group in Germany gave an excellent summary of the plant-associated compounds that have now been isolated and reported through late 2012 from endophytic microbes isolated from the “producing plant(s).” These included vincristine, camptothecin plus its 9-methoxy and 10-hydroxy derivatives, podophyllotoxin, hypericin and its probable biosynthetic precursor, emodin, azadirachtin A and B and some of the loline alkaloids (Aly et al., 2013).

To this excellent review should be added the following recent papers covering some of the compounds above and some unusual findings which give further direct evidence of fungal involvement. Thus, Ramesha et al. (2013) identified three endophytic fungi isolated from the fruit and seed regions of the plant *Miquelia dentata* Bedd which is reported to have very high concentrations of camptothecins in its seeds, as *Fomitopsis* sp., *Alternaria alternata*, and *Phomosis* sp. What is very intriguing is that in a paper a year later, the authors reported that, contrary to what they would have expected, these three fungi were inhibited by camptothecins, so there may well be negative feedback loops controlling production (Shweta et al., 2014).

#### Swainsonine

**Figure 2:**
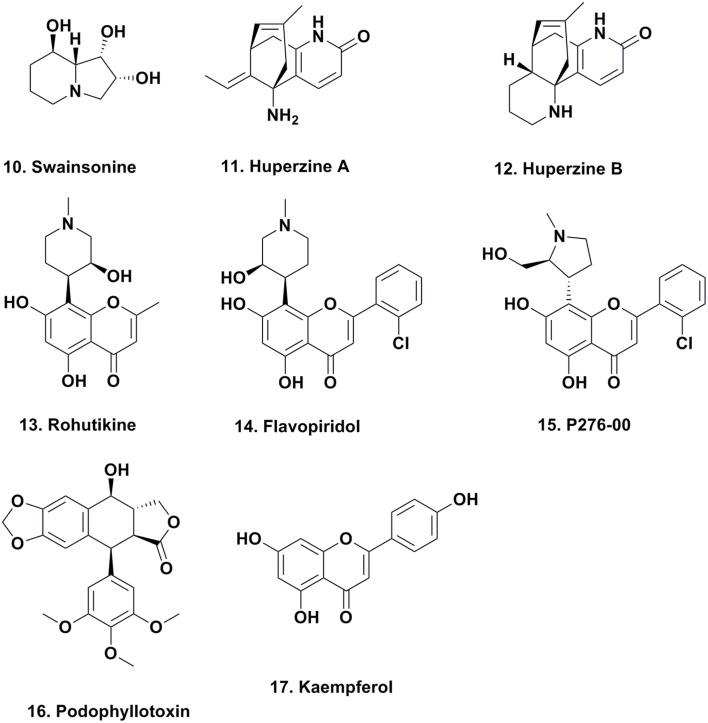
**Compounds from Endophytic Fungi**.

The relationship between fungal presence and swainsonine production was first published in 2003 (Braun et al., 2003) and very interestingly, the fungus, an *Undifilum* sp., was transferred by vertical transmission via the seed (Oldrup et al., 2010; Ralphs et al., 2011). Subsequently, in the last 3 years, three papers have been published that definitively prove that the compound swainsonine (Figure 2; **10**), the active component of “locoweed,” is in fact produced by endophytic fungi isolated from the producing plant. The paper published by Cook et al. (2013) covered the production of the alkaloid from a fungal endophyte in the seeds of *Ipomoea carnea*, and the abolition of production by treatment of the seeds with a fungicide, but production of other metabolites such as the calystegnines was unaltered.

Thus, removal of the fungus from the seed abolished production of the compound but other plant-derived metabolites were unaltered. This is a rather nice proof of the actual producer since without the fungus, the germinated plant did not produce swainsonine.

In the middle of 2013, the same group published details of the chemistry of swainsonine isolated this time from the original plant source of the alkaloid, the Australian-sourced *Swainsonia canescens* (Grum et al., 2013), and again, an endophytic fungus closely related to the genus *Undifilum* was the actual producer. In 2014, a follow-up paper from the same group (Cook et al., 2014) covered the production of the alkaloid from a variety of plant hosts and their associated fungi over wide geographic areas of the world.

#### Huperzine

Huperzines A and B (Figure 2; **11, 12**) are acetylcholinesterase inhibitors originally reported as part of Traditional Chinese Medicine (TCM) (Qin and Xu, 1998) isolated from *Huperzia serrata*. Huperzine A was originally synthesized as a racemic mixture and reported in 1990 with some definition of its pharmacological properties (Kozikowski et al., 1990), and later, of its binding to acetylcholinesterase (Raves et al., 1997). The material was launched as a nutraceutical and some clinical trials are still ongoing.

However, in 2014, two papers were published identifying an endophytic fungus isolated from *H. serrata* that produced the compound (Dong et al., 2014; Shu et al., 2014). The same year, another group demonstrated the ability of fungal endophytes also isolated from *H. serrata* to biotransform huperzine A to form bioactive sesquiterpenoid hybrids given the trivial name of Huptremules A–D. All of these hybrids however, were two orders of magnitude less active as AchE inhibitors compared to the parent compound (Ying et al., 2014). What is of interest however, is that these investigators did not discover the producing fungus referred to above. From the data provided, it is not certain if plants from the same geographic area were used, or if similar meteorological conditions applied in each case, as these are known to affect metabolites found in plants.

#### Rohutikine

Rohutikine (Figure 2; **13**) came into prominence as the model for the semisynthetic compound flavopiridol (Figure 2; **14**) which reached Phase II clinical trials in cancer and was heading for Phase III when Sanofi-Aventis discontinued development. In 2014, flavopiridol was licensed to Tolero Pharmaceuticals in Utah, USA who are planning to initiate Phase III studies in acute myelogenous leukemia.

Rohutikine was also the basis for Piramal's P276-00 (Figure 2; **15**) whose status is uncertain due to Piramal's recent cessation of small molecule drug discovery, though it was in clinical trials in the USA for cancer.

Initially the sources of rohutikine were *Amoora rohituka* and *Dysoxylum binectariferum*. It was later reported from *Schumanniophyton magnificum* and *S. problematicum*. Due to the therapeutic potential observed for rohutikine derivatives, there was a search for other producers including endophytes. In 2012 Mohana Kumara *et al* reported the production of rohutikine by fermentation of the endophytic fungus *Fusarium proliferatum* isolated from *D. binectariferum* (Mohana Kumara et al., 2012). In 2014 the same group reported that four other fungal species, three *Fusarium* isolates from *D. binectariferum* and one, *Gibberella fujikuroi* isolated from *A. rohituka*, also produced the compound on fermentation., They did make the point that the yield dropped on extended cultivation, though this may be due to the loss of as yet unknown co-factors (see discussion earlier on competitive fermentations and switching on of cryptic clusters) (Mohana Kumara et al., 2014).

#### Kaempferol

In a recent paper, Huang et al. (2014) described the isolation of endophytic fungi from surface sterilized rhizomes of the high-altitude plant *Sinopodophyllum hexandrum* Royal collected in the Taibai Mountains of China. These isolated fungi produced both podophyllotoxin and kaempferol (Figure 2; **16, 17**) on fermentation. The reason for looking at this particular plant/geographic area was the initial report by Ying (1979) that this plant produced both of the compounds. One fungus produced only kaempferol but another identified as *Mucor fragilis* Fresen. (Mucoraceae) produced both compounds and was deposited in the China Center for Type Culture Collection as M 205032. The authors suggested horizontal gene transfer (HGT) from the plant to the fungus but equally the fungus, under cryptic cluster control (*vide infra*), could be the source for the plant to use as protective agents against attack.

#### Plant-derived compounds from epiphytes/endophytes (and/or root associated microbes)

Due to the differences in definition by multiple authors as to epiphyte and endophyte, when a compound is reported from microbes that are not “within” the tissues of the plant we have discussed them in this section. As information is published, the actual producer may “move” within these definitions as shown below for maytansine and the ergot alkaloids.

#### Maytansine

**Figure 3:**
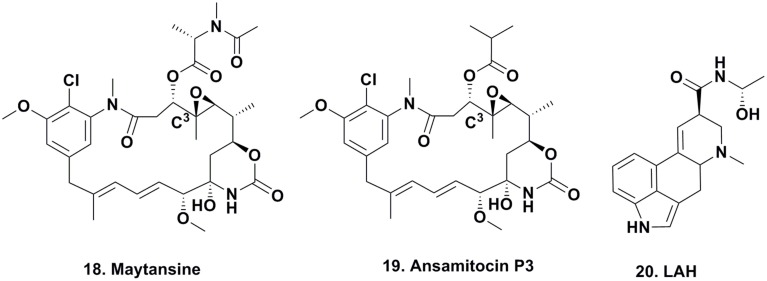
**Compounds from Epiphytic and “Endophytic” Microbes**.

For many years, maytansine (Figure 3; **18**) and congeners were thought to be exclusively plant-derived secondary metabolites. Maytansine was first reported by Kupchan et al. (1972) isolated in very low yield from *Maytenus ovatus* collected in Ethiopia, and later isolated from *M. buchananii* and *Putterlickia verrucosa*. The compound also exhibited anti-parasitic and antimicrobial activity, and based on maytansine exhibiting potent cytotoxic activity against human KB cells, as well as several other cancer cell lines, researchers became interested in using this pharmacophore for the treatment of cancer. Though total syntheses were reported by the Meyers (Meyers and Shaw, 1974) and Corey research groups (Corey et al., 1980), these syntheses were multi-step, time- and labor-intensive, and impractical for large-scale synthesis for clinical trials, so large-scale extraction processes were used to obtain enough material for clinical trials.

Since maytansine was a 19-membered, halogenated ansamycin, an unusual structure for a plant secondary metabolite, but a chemotype that is commonly produced by eubacteria, and was found to be present in some but not all individual *P. verrucosa* plants, a search commenced for microorganisms (fungal or eubacterial endophytes) that could produce its core structure.

In 1977, investigators at Takeda Industries in Japan reported the discovery of ansamitocins P-0, P-1, P-2, P-3 (Figure 3; **19**), P-3′, and P-4, which are maytansine-like derivatives with either an ester or hydroxyl moiety at C^3^, from two subspecies of *Nocardia* (subsequently renamed as *Actinosynenna pretiosum*) isolated from the *Carex* species of grassy plants (Higashide et al., 1977). Because the only difference between maytansine and ansamitocin P-3 is the ester moiety at C^3^, and none of the biosynthetic genes leading to the production of maytansine had been found in the plant host (Yu et al., 2012), researchers speculated that the P-3 precursor was produced by an endophyte or symbiont in the rhizosphere, followed by uptake of the bacterial metabolite and converted into maytansine.

This hypothesis seemed plausible, since several ansamitocins are produced by eubacteria, higher plants, and mosses, contradicting the common evolutionary theory that natural products are produced by taxonomically-related organisms. Wings and coworkers grew axenic cultures of *P. verrucosa* and could not amplify genes involved in maytansine biosynthesis, and a maytansine-producing eubacterium could not be cultured outside of its natural habitat (Wings et al., 2013). By using molecular techniques such as rDNA sequencing and single strand conformation polymorphism, they identified that the *A. pretiosum* ssp. *auranticum* eubacterium present in the rhizosphere of the plant is involved in maytansine biosynthesis. Whether this is an epiphyte or a root-associate endophyte is not yet fully elucidated.

Based on rDNA sequence analysis, the *A. pretiosum* ssp. *auranticum* eubacterium had the identical 16S rDNA sequence as that amplified from the DNA of a maytansine-producing *P. verrucosa* plant (Wings et al., 2013). Other non-maytansine producing *P. verrucosa* plants lacked this 16S rDNA sequence. These data are consistent with the absence of maytansine in cell cultures derived from maytansine-producing *P. verrucosa* plants as well as greenhouse grown *Maytenus* sp., and *Putterlickia* sp., plants and their corresponding cell cultures (Wings et al., 2013).

Mounting evidence has shown that the microorganisms in the rhizosphere of plants in different environments as well as those in non-rhizosphere communities in the surrounding soil appear to differ (Gunatilaka, 2006). This may explain why maytansine is found in mosses and higher plants. However, nominally ansamitocin-producing plants have been speculated to contribute to the structural diversity of ansamitocins via infection of their root system because only two known ansamitocins have been found in eubacteria, while there are 22 known in plants (Wings et al., 2013).

In 2014, the debate as to whether the ansamitocin derivatives produced in the rhizosphere were subsequently transported into the plant and then trans-esterified to produce maytansine from ansamitocin P3, was decided in favor of the production of maytansine by a consortium of microbes in the rhizosphere of the plants *Putterlickia verrucosa* and *P. retrospinosa*, though the exact organism(s) performing the reaction are not yet identifiable (Kusari et al., 2014c). Thus, the materials found in specific areas may well be the products of multiple interactions outside of and within the “nominal plant producer.”

#### Ergot alkaloids

There is one well defined series of compounds that are considered to be produced via epiphytes that has been known for centuries; the production of the ergot alkaloids such as lysergic acid α-hydroxyethylamide (Figure 3; **20**) due to the contamination of rye by the fungus *Claviceps purpurea*.

In a recent publication, Beaulieu *et al* reported on the expansion of biosynthetic capabilities beyond *Claviceps* species, including bacterial and fungal symbionts depending upon the host plant (Beaulieu et al., 2013). What is significant, though it had been known for a reasonable amount of time, was the vertical transmission of the epiphyte in the seeds of the infected plant, and they described the allocation of these alkaloids during the early ontology of Morning Glory plants (*Ipomoea* species), though the fungus in these cases was close to a *Periglandula*-like species. As mentioned earlier in this review, *I. carnea* was reported to produce swainsonine via a vertically transmitted microbe as well.

In 2014, Hodgson et al. (2014) reported that vertical transmission of fungal endophytes is widespread in “forbs” (also known as herbs or Phorbs) which are defined by the USDA (United States Department of Agriculture) as:

“Vascular plant without significant woody tissue above or at the ground. Forbs and herbs may be annual, biennial, or perennial but always lack significant thickening by secondary woody growth and have perennating buds borne at or below the ground surface. In plants, graminoids are excluded but ferns, horsetails, lycopods, and whisk-ferns are included. (http://plants.usda.gov/growth_habits_def.html)”

Thus, the phenomenon of such vertical transmission is not rare but an integral part of how a plant may recruit defensive measures. As to whether these are co-evolution, horizontal gene transfer or mutualistic survival methodologies, one can make a choice, but it is now becoming quite evident that such interactions between plants and microbes are very common and not rare occurrences.

## Compounds from fungal-bacterial interactions

### Rhizoxin and derivatives

**Figure 4:**
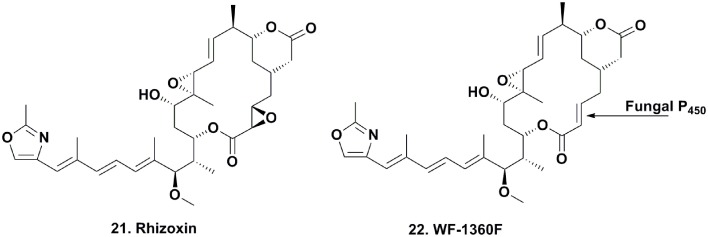
**Compounds from Fungal-endophytic Bacterial Association**.

Rhizoxin (Figure 4; **21**) was reported in 1984 by Iwasaki et al. (1984) from a *Rhizopus* species that caused rice blight and its antitumor activity was then reported by Tsuruo et al. (1986). It entered clinical trials as a tubulin interactive agent but did not proceed beyond Phase II due to a lack of significant responses in patients (Hanauske et al., 1996).

Many chemists used total synthesis to make rhizoxin and several derivatives (Nakada et al., 1993; Hong and White, 2004). In the early 2000s, Partida-Martinez and Hertweck began to investigate the biosynthesis of the compound in *Rhizopus* via fermentation, and rapidly discovered that rhizoxin was not a fungal metabolite, but rather a product of an eubacterial endosymbiont *Burkholderia* sp. On isolation and purification of this bacterium, they demonstrated that the organism contained the biosynthetic genes involved in the production of rhizoxin (Partida-Martinez and Hertweck, 2005, 2007).

These observations were consistent with four *Rhizopus* species producing rhizoxin and two species that did not, when collected in diverse geographical areas. Furthermore, laser microscopic observations of *Rhizopus* sp. mycelium stained with a mixture of bacteria-specific dyes revealed the appearance of a high number of live endosymbiotic eubacteria within fungal cells. Notably, when *Rhizopus* sp. was cultured in the absence of the *Burkholderia* endosymbiont, rhizoxin was not produced. However, when the *Burkholderia* sp. was isolated from the fungus and cultured in the absence of *Rhizopus* sp., rhizoxin and potent cytotoxic derivatives (1000–10,000 times more active against K-562 leukemia cells) were produced (Scherlach et al., 2006). Interestingly, the isolated eubacterial endosymbiont lost its ability to produce these metabolites over time, but rhizoxin increased upon the reintroduction of *Rhizopus* sp. into cultures. The authors speculated that the decrease in rhizoxin was most likely due to the down-regulation of its biosynthetic genes in the absence of *Rhizopus* sp.

Deletion of a *Burkholderia* p450 gene involved in rhizoxin biosynthesis produced di-desepoxy rhizoxin derivatives, but whether this gene was involved in catalyzing the formation of both epoxide moieties in rhizoxin was unclear (Scherlach et al., 2012). The epoxidation steps were also determined to be oxygen independent.

To elucidate the biosynthetic steps required to install the epoxide moieties, the authors used two different *Burkholderia*-*Rhizopus* associations from different regions of the world that either produced rhizoxin or the monoepoxide derivative WF-1360F (Figure 4; **22**). Using these combinations, they “switched” the symbiotic associations by cross-infecting each endosymbiotic-free *R. microporus* fungus with the endosymbiotic eubacterium of the other fungus. Interestingly, the symbiotic association that previously produced rhizoxin produced WF-1360F, whereas the other association produced rhizoxin. Thus, these results led the authors to revise their proposed mechanism of rhizoxin biosynthesis in the 2005 Nature paper (Partida-Martinez and Hertweck, 2005).

These events are most likely triggered by chemical signals. These are probably produced via the symbiotic phytotoxin production resulting from the strain-specific association of *Burkholderia* sp. and *Rhizopus* sp. In addition, these may be further influenced by plant interactions upon infection of the rice seedlings.

Thus, the vertically transmitted eubacterial intracellular symbiont of *Rhizopus* sp. delivers WF-1360F to the host fungus, which is then involved in catalyzing the epoxidation of the WF-1360F to give rhizoxin. This is a more potent phytotoxin that plays an essential role in the vegetative spore formation of the fungus containing the endosymbiont, most likely for colonizing rice (Partida-Martinez et al., 2007). In this unparalleled tripartite relationship, both the pathogenic fungus and endosymbiont benefit by gaining access to nutrients that are released once the phytopathogenic fungus colonizes the roots of *Oryza sativa*.

### Insect-microbe interactions

We will discuss two of the many potential examples of this type of interaction. Though many are postulated, in the two examples given, the interactions have been characterized as harnessing the metabolites produced as protective factors of benefit to the arthropod hosts.

### Dentigerumycin production

**Figure 5:**
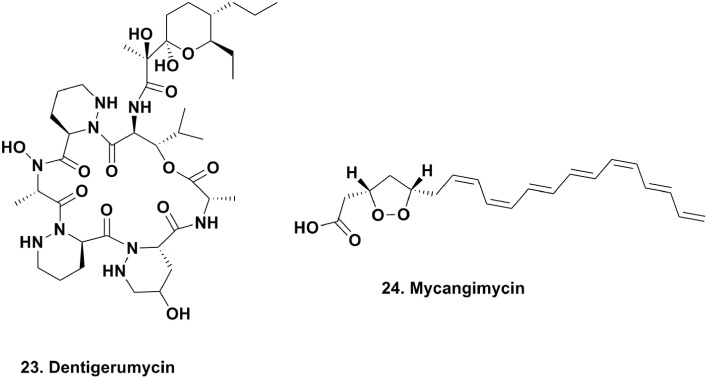
**Compounds from Microbe-Insect Association**.

The seminal work published on dentigerumycin (Figure 5; **23**) by Oh and coworkers, demonstrated how fungus-growing ants and actinobacteria work together to produce a specific toxin that specifically eliminates specialized fungal parasites (Oh et al., 2009a). In 2001, the eubacterium *Pseudonocardia* sp., fungal isolates (used as a food source for these attine ants), and the parasitic fungus *Escovopsis* sp. were isolated from the nest of the ant *Apterostigma dentigerum* in Gamboa, Panama. The *Pseudonocardia* sp., isolated from the ant cuticle, was observed to strongly inhibit *Escovopsis* sp. from the same ant colony, while the fungal isolates were resistant to this bacterium.

The active component isolated from the *Pseudonocardia* sp. was the depsipeptide dentigerumycin, which contained highly unusual amino acid residues, such as piperazic acid, γ-hydroxypiperazic acid, β-hydroxyleucine, *N*-hydroxyleucine, and a polyketide-derived side chain linked to a pyran ring. Dentigerumycin inhibited the growth of the *Escovopsis* sp., as well as *Candida albicans* strains, including the amphotericin-resistant version ATCC200955, in liquid culture assays.

Thus, the symbiosis between *Pseudonocardia* sp. and fungus-farming ants is an example of novel ways ants have evolved to protect the fungal cultivar from “garden parasites.” Notably, the authors speculated that the eubacterial mediator *Pseudonocardia* sp. and the fungus *Escovopsis* sp. will undergo antagonistic coevolution, such that new eubacterial metabolites will target resistant *Escovopsis* sp. Such evolutionary processes may well play major roles in the continuous production of new, diverse secondary metabolites from mutualistic interactions.

### Mycangimycin production by beetle symbionts

Scott and coworkers reported the existence of chemically-mediated protection supplied by a eubacterial source against the fungal antagonist, *Ophiostoma minus*, of the fungal food source (*Entomocorticium* sp. A) required for the development of Southern pine beetle (*Dendroctonus frontalis*) larvae (Scott et al., 2008). Adult beetles harbor *Entomocorticium* sp. A in a specialized compartment, make holes in the barks of trees, deposit larvae within the bark and phloem of trees, and inoculate them with this fungus. This process can be challenged by a parasitic fungus that can outcompete *Entomocorticum* sp. A, ultimately disrupting beetle larvae development.

As part of the beetle's defense mechanism, its specialized compartment harboring food is also a source of different species of actinomycetes, which are also deposited with *Entomocorticum* sp. A. The authors were able to demonstrate the antifungal activity of one actinomycete morphotype against *O. minus* with an MIC of 1.0 μM, which was 19 times more susceptible than *Entomocorticum* sp. A (MIC, 19.0 μM). The active antifungal agent was determined to be the linear 20-carbon polyunsaturated peroxide, mycangimycin (Figure 5; **24**) (Oh et al., 2009b). This compound also exhibited potent antifungal activity against *C. albicans, C. albicans* ATCC 10231, *C. albicans* ATCC 200955, and *Saccharomyces cerevisiae*, with MIC values ranging between 0.7 and 1.4 μM. The following year, there was a report of a free-living actinomycete producing the same material from an Egyptian soil sample (Atta, 2010).

The basic scaffold of mycangimycin resembles those of some known antimalarial agents, and when assayed against *Plasmodium falciparum*, the compound exhibited antimalarial activity with an EC_50_ of 17 ng/ml, which is comparable to other antimalarial drugs with EC_50_ values close to 10 ng/ml. More studies need to be completed to determine the mechanism of action of mycangimycin, as well as whether it possesses other biological properties. However, this is a good example of how specialized small molecules that serve as mediators within mutualistic interactions can also function as new therapeutics.

## In conclusion

In this short review, we have attempted to demonstrate that in all kingdoms of life, microbes may play a role in the production of secondary metabolites in “higher hosts.” Does this mean that we are saying that “ALL secondary metabolites irrespective of the higher host are microbial in origin”? The current answer overall is NO for plants, but in the marine environment, the pendulum may well be swinging toward “YES.”

With the recognition of chalcone synthases being present in marine microbes, it might be of interest to note that 262 terpene synthase genes have recently been identified from terrestrial microbial genome sequences by workers at the Kitasato Institute and we have inserted their conclusions in the following paragraph.

“Terpenes are generally considered to be plant or fungal metabolites, although a small number of odoriferous terpenes of bacterial origin have been known for many years. Recently, extensive bacterial genome sequencing and bioinformatic analysis of deduced bacterial proteins using a profile based on a hidden Markov model have revealed 262 distinct predicted terpene synthases. Although many of these presumptive terpene synthase genes seem to be silent in their parent microorganisms, controlled expression of these genes in an engineered heterologous Streptomyces host has made it possible to identify the biochemical function of the encoded terpene synthases. Genes encoding such terpene synthases have been shown to be widely distributed in bacteria and represent a fertile source for discovery of new natural products” (Yamada et al., 2015).

Thus, can one now claim that terpene synthases and chalcone synthases are all from eukaryotes in the future?

However, when one investigates the relationships between hosts and microbes in marine and terrestrial environments, it is striking that the types of interaction, in particular those leading to secondary metabolites are many and complex. They are not as simple as saying that “X” is a plant metabolite and “Y” comes from a marine invertebrate. Yes, each was isolated from a specific “host” but the question as to what combination of events produced the compound is no longer simple to answer.

Investigators have to take into account that as yet uncultivated microbes are probably the major sources of these interactions, and that simple culturing techniques may not be adequate to identify the range of potential interactions. It is not a one host/one microbe style of interaction but probably involves many interactions between microbes and the host, not just a simple one to one relationship. When one then has to consider “cryptic gene clusters and their control (*cf* the terpene synthase discussion above).”

What has to also be recognized, and it is alluded to in some of the examples given above, is that in Nature, microbes are not “singletons,” they are part of essential consortia. Many examples are available to demonstrate this, with one being the mixed cultures inside a very protective biofilm that is the essential part of the metabolism of phosphates in sewage plants. Similar collections of microbes are present in soils and marine invertebrates, and also in vertebrates in general, as all one has to do is to look at the information now appearing on the human microbiome.

To finish and to give an idea of the magnitude of the processes potentially involved, the very recent review by Charlop-Powers et al. (2015) should be consulted to see the magnitude of secondary metabolites that are potentially present, and then to think of the vast number of interactions, yet to be discovered.

### Conflict of interest statement

The authors declare that the research was conducted in the absence of any commercial or financial relationships that could be construed as a potential conflict of interest.

## References

[B1] Aly A. H. Debbab A. Proksch P. (2013). Fungal endophytes—secret producers of bioactive plant metabolites. Pharmazie 68, 499–505. 23923629

[B2] Arai T. Takahashi K. Kubo A. (1977). New antibiotics, Saframycins A, B, C, D and E. J. Antibiot. 30, 1015–1018. 10.7164/antibiotics.30.1015 591455

[B3] Arai T. Takahasi K. Ishiguro K. Mikami Y. (1980). Some chemotherapeutic properties of two new antitumor antibiotics saframycins A and C. Gann 71, 790–796. 7274625

[B4] Atta H. M. (2010). Production, purification, physico-chemical characteristics and biological activities of an antifungal antibiotic produced by *Streptomyces antibioticus*, AZ-Z710. Amer. Euras. J. Sci. Res. 5, 39–49.

[B5] Beaulieu W. T. Panaccione D. G. Hazekamp C. S. McKee M. C. Ryan K. L. Clay K. (2013). Differential allocation of seed-borne ergot alkaloids during early ontogeny of Morning Glories (Convolvulaceae). J. Chem. Ecol. 39, 919–930. 10.1007/s10886-013-0314-z 23835852

[B6] Bertrand S. Azzollini A. Schumpp O. Bohni N. Schrenzel J. Monod M.. (2014). Multi-well fungal co-culture for *de novo* metabolite-induction in time-series studies based on untargeted metabolomics. Mol. Biosyst. 10, 2289–2298. 10.1039/C4MB00223G 24948000

[B7] Braun K. Romero M. Liddell C. Creamer R. (2003). Production of swainsonine by fungal endophytes of locoweed. Mycol. Res. 107, 980–988. 10.1017/S095375620300813X 14531620

[B8] Charlop-Powers Z. Owen J. G. Reddy B. V. B. Ternei M. A. Guimarães D. O. de Frias U. A.. (2015). Global biogeographic sampling of bacterial secondary metabolism. Elife 4:e05048. 10.7554/eLife.05048 25599565 PMC4383359

[B9] Cook D. Beaulieu W. T. Mott I. W. Riet-Correa F. Gardner D. R. Grum D.. (2013). Production of the alkaloid Swainsonine by a fungal endosymbiont of the ascomycete order *Chaetothyriales* in the host *Ipomoea carnea*. J. Agric. Food Chem. 61, 37973803. 10.1021/jf4008423 23547913

[B10] Cook D. Gardner D. R. Pfister J. A. (2014). Swainsonine-containing plants and their relationship to endophytic fungi. J. Agric. Food Chem. 62, 7326–7334. 10.1021/jf501674r 24758700

[B11] Corey E. J. Weigel L. O. Chamberlin A. R. Cho H. Hua D. H. (1980). Total synthesis of maytansine. J. Am. Chem. Soc. 102, 6613–6615. 10.1021/ja00541a064

[B12] Cuevas C. Francesch A. (2009). Development of Yondelis® (trabectedin, ET-743). A semisynthetic process solves the supply problem. Nat. Prod. Rep. 26, 322–337. 10.1039/b808331m 19240944

[B13] Cuevas C. Francesch A. Galmarini C. M. Aviles P. Munt S. (2012). Ecteinascidin-743 (Yondelis^(R)^), Aplidin^(R)^, and Irvalec^(R)^, in Anticancer Agents from Natural Products, 2nd Edn., eds Cragg G. M. Kingston D. G. I. Newman D. J. (Boca Raton, FL: Taylor and Francis), 291–316.

[B14] Dong L.-H. Fan S.-W. Ling Q.-Z. Huang B.-B. Wei Z.-J. (2014). Indentification of huperzine A-producing endophytic fungi isolated from *Huperzia serrata*. World J. Microbiol. Biotech. 30, 1011–1017. 10.1007/s11274-013-1519-6 24129696

[B15] Flahive E. Srirangam J. (2012). The dolastatins: novel antitumor agents from *Dolabella auricularia,”* in Anticancer Agents from Natural Products, 2nd Edn. eds Cragg G. M. Kingston D. G. I. Newman D. J. (Boca Raton, FL: Taylor and Francis), 263–289.

[B16] Frincke J. M. Faulkner D. J. (1982). Antimicrobial metabolites of the sponge *Reniera* sp. J. Am. Chem. Soc. 104, 265–269. 10.1021/ja00365a0483829146

[B17] Grum D. S. Cook D. Baucom D. Mott I. W. Gardner D. R. Creamer R.. (2013). Production of the alkaloid Swainsonine by a fungal endophyte in the host *Swainsona canescens*. J. Nat. Prod. 76, 1984–1988. 10.1021/np400274n 24053110

[B18] Gunatilaka A. A. L. (2006). Natural products from plant-associated microorganisms: distribution, structural diversity, bioactivity, and implications of their occurrence. J. Nat. Prod. 69, 509–526. 10.1021/np058128n 16562864 PMC3362121

[B19] Guo F. Xiang S. Li L. Wang B. Rajasärkkä J. Gröndahl-Yli-Hannuksela K.. (2015). Targeted activation of silent natural product biosynthesis pathways by reporter-guided mutant selection. Metab. Eng. 28, 134–142. 10.1016/j.ymben.2014.12.006 25554073

[B20] Hanauske A. R. Catimel G. Aamdal S. ten Bokkel Huinink W. Paridaens R. Pavlidis N.. (1996). Phase II clinical trials with rhizoxin in breast cancer and melanoma. The EORTC Early Clinical Trials Group. Br. J. Cancer 73, 397–399. 10.1038/bjc.1996.68 8562349 PMC2074418

[B21] Heinig U. Scholtz S. Jennewein S. (2013). Getting to the bottom of Taxol biosynthesis by fungi. Fung. Divers. 60, 161–170. 10.1007/s13225-013-0228-7

[B22] Helfrich E. J. N. Reiter S. Piel J. (2014). Recent advances in genome-based polyketide discovery. Curr. Opin. Biotech. 29, 107–115. 10.1016/j.copbio.2014.03.004 24762576

[B23] Higashide E. Asai M. Ootsu K. Tanida S. Kozai Y. Hasegawa T.. (1977). Ansamitocin, a group of novel maytansinoid antibiotics with antitumour properties from *Nocardia*. Nature 270, 721–722. 10.1038/270721a0 593392

[B24] Hodgson S. Cates C. Hodgson J. Morley N. J. Sutton B. C. Gange A. C. (2014). Vertical transmission of fungal endophytes is widespread in forbs. Ecol. Evol. 4, 1199–1208. 10.1002/ece3.953 24834319 PMC4020682

[B25] Holt T. G. (1986). The Isolation and Structural Characterization of the Ecteinascidins. Ph.D. thesis, University of Illinois at Urbana-Champaign, Urbana-Champaign.

[B26] Hong J. White J. D. (2004). The chemistry and biology of rhizoxins, novel antitumor macrolides from *Rhizopus chinensis*. Tetrahedron 60, 5653–5681. 10.1016/j.tet.2004.04.032

[B27] Huang J.-X. Zhang J. Zhang X.-R. Zhang K. Zhang X. He X.-R. (2014). Mucor fragilis as a novel source of the key pharmaceutical agents podophyllotoxin and kaempferol. Pharm. Biol. 52, 1237–1243. 10.3109/13880209.2014.885061 24863281

[B28] Iwasaki S. Kobayashi M. Furukawa J. Namikoshi M. Okuda S. Sato Z.. (1984). Studies on macrocyclic lactone antibiotics. VII. Structure of a phytotoxin rhizoxin produced by *Rhizopus chinensis*. J. Antibiot. 37, 354–362. 10.7164/antibiotics.37.354 6547134

[B29] Kozikowski A. P. Yamada F. Tang X. C. Hanin I. (1990). Synthesis and biological evaluation of (±)-*Z*-huperzine-A. Tet. Lett. 31, 6159–6162. 10.1016/S0040-4039(00)97013-8

[B30] Kupchan S. M. Komoda Y. Court W. A. Thomas G. J. Smith R. M. Karim A.. (1972). Tumor inhibitors. LXXIII. Maytansine, a novel antileukemic ansa macrolide from *Maytenus ovatus*. J. Am. Chem. Soc. 94, 1354–1356. 10.1021/ja00759a054 5062169

[B31] Kusari S. Lamsho M. Kusari P. Gottfried S. Zuhlke S. Louven K.. (2014c). Endophytes are hidden producers of maytansine in *Putterlickia* roots. J. Nat. Prod. 77, 2577–2584. 10.1021/np500219a 25478947

[B32] Kusari S. Pandey S. P. Spiteller M. (2013). Untapped mutualistic paradigms linking host plant and endophytic fungal production of similar bioactive secondary metabolites. Phytochemistry 91, 81–87. 10.1016/j.phytochem.2012.07.021 22954732

[B33] Kusari S. Singh S. Jayabaskaran C. (2014a). Biotechnological potential of plant-associated endophytic fungi: hope versus hype Trends Biotechnol. 32, 297–303. 10.1016/j.tibtech.2014.03.009 24703621

[B34] Kusari S. Singh S. Jayabaskaran C. (2014b). Rethinking production of Taxol^(R)^ (paclitaxel) using endophyte biotechnology. Trends Biotechnol. 32, 304–311. 10.1016/j.tibtech.2014.03.011 24810040

[B35] Lee J. Currano J. N. Carroll P. J. Joullié M. M. (2012). Didemnins, tamandarins and related natural products. Nat. Prod. Rep. 29, 404–424. 10.1039/c2np00065b 22270031

[B36] Li L. Deng W. Song J. Ding W. Zhao Q.-F. Peng C.. (2008). Characterization of the saframycin A gene cluster from *Streptomyces l avendulae* NRRL 11002 revealing a nonribosomal peptide synthetase system for assembling the unusual tetrapeptidyl skeleton in an iIterative manner. J. Bact. 190, 251–263. 10.1128/JB.00826-07 17981978 PMC2223732

[B37] Li Y. C. Tao W. Y. Cheng L. (2009). Paclitaxel production using co-culture of *Taxus* suspension cells and paclitaxel-producing endophytic fungi in a co-bioreactor. Appl. Microbiol. Biotechnol. 83, 233–239. 10.1007/s00253-009-1856-4 19172266

[B38] Luesch H. Harrigan G. G. Goetz G. Horgen F. D. (2002). The cyanobacterial origin of potent anticancer agents originally isolated from sea hares. Curr. Med. Chem. 9, 1791–1806. 10.2174/0929867023369051 12369878

[B39] Ma H. Lu Z. Liu B. Qiu Q. Liu J. (2013). Transcriptome analyses of a Chinese hazelnut species *Corylus mandshurica*. BMC Plant Biol. 13:152. 10.1186/1471-2229-13-152 24093758 PMC3819738

[B40] Meyers A. Shaw C.-C. (1974). Studies directed toward the total synthesis of maytansine. The preparation and properties of the carbinolamide moiety. Tetrahedron Lett. 15, 717–720. 10.1016/S0040-4039(01)82313-3

[B41] Mohana Kumara P. Soujanya K. N. Ravikanth G. Vasudeva R. Ganeshaiah K. N. Shaanker R. U. (2014). Rohitukine, a chromone alkaloid and a precursor of flavopiridol, is produced by endophytic fungi isolated from *Dysoxylum binectariferum* Hook.f and *Amoora rohituka* (Roxb). *Wight Arn*. Phytomed. 21, 541–546. 10.1016/j.phymed.2013.09.019 24215673

[B42] Mohana Kumara P. Zuehlke S. Priti V. Ramesha B. T. Shweta S. Ravikanth G.. (2012). *Fusarium proliferatum* an endophytic fungus from *Dysoxylum binectariferum* Hook.f, produces rohutikine, a chromane alkaloid possessing anti-cancer activity. Anton. Van Leeuwen. 101, 323–329. 10.1007/s10482-011-9638-2 21898150

[B43] Moore B. S. Hertweck C. Hopke J. N. Izumikawa M. Kalaitzis J. A. Nilsen G.. (2002). Plant-like biosynthetic pathways in bacteria: from benzoic acid to chalcone. J. Nat. Prod. 65, 1956–1962. 10.1021/np020230m 12502351

[B44] Moss C. Green D. H. Perez B. Velasco A. Henriquez R. McKenzie J. D. (2003). Intracellular bacteria associated with the ascidian *Ecteinascidia turbinata*: phylogenic and *in situ* hybridization analysis. Mar. Biol. 143, 99–110. 10.1007/s00227-003-1060-5

[B45] Nakada M. Kobayashi S. Iwasaki S. Ohno M. (1993). The first total synthesis of the antitumor macrolide rhizoxin: synthesis of the key building blocks. Tetrahedron Lett. 34, 1035–1038. 10.1016/S0040-4039(00)77485-59306725

[B46] Nakao Y. Shiroiwa T. Murayama S. Matsunaga S. Goto Y. Matsumoto Y. (2004). Identification of Renieramycin A as an antileishmanial substance in a marine sponge *Neopetrosia* sp. Mar. Drugs 2, 55–62. 10.3390/md202055

[B47] Newman D. J. Cragg G. M. (2010). Natural products as drugs and leads to drugs: the historical perspective, in Natural Product Chemistry for Drug Discovery, eds Buss A. D. Butler M. S. (Cambridge: Royal Society of Chemistry), 3–27.

[B48] Newman D. J. Cragg G. M. (2014). Marine-sourced anti-cancer and cancer pain control agents in clinical and late preclinical development. Mar. Drugs 12, 255–278. 10.3390/md12010255 24424355 PMC3917273

[B49] Oh D.-C. Poulsen M. Currie C. R. Clardy J. (2009a). Dentigerumycin: a bacterial mediator of an ant-fungus symbiosis. Nat. Chem. Biol. 5, 391–393. 10.1038/nchembio.159 19330011 PMC2748230

[B50] Oh D.-C. Scott J. J. Currie C. R. Clardy J. (2009b). Mycangimycin, a polyene peroxide from a mutualist *Streptomyces* sp. Org. Lett. 11, 633–636. 10.1021/ol802709x 19125624 PMC2640424

[B51] Oldrup E. McLain-Romero J. Padilla A. Moya A. Gardner D. R. Creamer R. (2010). Localization of endophytic *Undifilum* fungi in locoweed seed and influence of environmental parameters on a locoweed *in vitro* culture system. Botany 88, 512–521. 10.1139/B10-026

[B52] Partida-Martinez L. P. Hertweck C. (2005). Pathogenic fungus harbours endosymbiotic bacteria for toxin production. Nature 437, 884–888. 10.1038/nature03997 16208371

[B53] Partida-Martinez L. P. Hertweck C. (2007). A gene cluster encoding rhizoxin biosynthesis in “Burkholderia rhizoxina”, the bacterial endosymbiont of the fungus Rhizopus microsporus. Chem. Bio. Chem. 8, 41–45. 10.1002/cbic.200600393 17154220

[B54] Partida-Martinez L. P. Monajembashi S. Greulich K.-O. Hertweck C. (2007). Endosymbiont-dependent host reproduction maintains bacterial-fungal mutualism. Curr. Biol. 17, 773–777. 10.1016/j.cub.2007.03.039 17412585

[B55] Perez-Matos A. E. Rosado W. Govind N. S. (2007). Bacterial diversity associated with the Caribbean tunicate *Ecteinascidia turbinata*. Anton. Van Leeuwen. 92, 155–164. 10.1007/s10482-007-9143-9 17265101

[B56] Piel J. (2006). Bacterial symbionts: prospects for the sustainable production of invertebrate- derived pharmaceuticals. Curr. Med. Chem. 13, 39–50. 10.2174/092986706775197944 16457638

[B57] Qin G.-W. Xu R.-S. (1998). Recent advances on bioactive natural products from Chinese medicinal plants. Med. Res. Rev. 18, 375–382. 9828038 10.1002/(sici)1098-1128(199811)18:6<375::aid-med2>3.0.co;2-8

[B58] Ralphs M. H. Cook D. Gardner D. R. Grum D. S. (2011). Transmission of the locoweed endophyte to the next generation of plants. Fungal Ecol. 4, 251–255. 10.1016/j.funeco.2011.03.00111359703

[B59] Ramesha B. T. Suma H. K. Senthilkumar U. Priti V. Ravikanth G. Vasudeva R.. (2013). New plant sources of the anti- cancer alkaloid, camptothecine from the Icacinaceae taxa, India. Phytomedicine 20, 521–527. 10.1016/j.phymed.2012.12.003 23474217

[B60] Rath C. M. Janto B. Earl J. Ahmed A. Hu F. Z. Hiller L.. (2011). Meta-omic characterization of the marine invertebrate microbial consortium that produces the chemotherapeutic natural product et-743. ACS Chem. Biol. 6, 1244–1256. 10.1021/cb200244t 21875091 PMC3220770

[B61] Raves M. Harel M. Pang Y. Silman I. Kozikowski A. Sussman J. (1997). Structure of acetylcholinesterase complexed with the nootropic alkaloid, (-)-huperzine A. Nat. Struct. Biol. 4, 57–63. 10.1038/nsb0197-57 8989325

[B62] Rinehart K. Holt T. G. Fregeau N. L. Stroh J. G. Kiefer P. A. Sun F. (1990). Ecteinascidins 729, 743, 745, 759A, 759B and 770: potent antitumor agents from the Caribbean tunicate *Ecteinascidia turbinata*. J. Org. Chem. 55, 4512–4515. 10.1021/jo00302a007

[B63] Rinehart K. L. Kishore V. Nagarajan S. Lake R. J, Gloer J. B. Bozich F. A. (1987). Total synthesis of Didemnin-A, Didemnin-B, and Didemnin-C. J. Am. Chem. Soc. 109, 6846–6848. 10.1021/ja00256a046

[B64] Scherlach K. Busch B. Lackner G. Paszkowski U. Hertweck C. (2012). Symbiotic cooperation in the biosynthesis of a phytotoxin. Angew. Chem. Int. Ed. 124, 9753–9756. 10.1002/ange.201204540 22915379

[B65] Scherlach K. Partida-Martinez L. P. Dahse H.-M. Hertweck C. (2006). Antimitotic rhizoxin derivatives from a cultured bacterial endosymbiont of the rice pathogenic fungus *Rhizopus microsporus*. J. Am. Chem. Soc. 128, 11529–11536. 10.1021/ja062953o 16939276

[B66] Scott J. J. Oh D.-C. Yuceer M. C. Klepzig K. D. Clardy J. Currie C. R. (2008). Bacterial protection of beetle-fungus mutualism. Science 322, 63. 10.1126/science.1160423 18832638 PMC2761720

[B67] Service R. F. (2000). Hazel trees offer a new source of cancer drug. Science 288, 1609–1610. 10.1126/science.288.5463.27a 10766629

[B68] Shu S. Zhao X. Wang W. Zhang G. Cosoveanu A. Ahn Y.. (2014). Identification of a novel endophytic fungus from *Huperzia serrata* which produces huperzine A. World J. Microbiol. Biotech. 30, 3101–3109. 10.1007/s11274-014-1737-6 25212543

[B69] Shweta S. Shivanna M. B. Gurumurthy B. R. Shaanker U. Santhosh Kumar T. R. Ravikanth G. (2014). Inhibition of fungal endophytes by camptothecine produced by their host plant, *Nothapodytes nimmoniana* (Grahm) Mabb. (Icacinaceae). Curr. Sci. 107, 994–1000.

[B70] Sigel M. M. Wellham L. L. Lichter W. Dudeck L. E. Gargus J. L. Lucas L. H. (1970). Food-drugs from the Sea: Proceedings 1969. Washington, DC: Marine Technology Society.

[B71] Soliman S. S. M. Raizada M. N. (2013). Interactions between co-habitating fungi elicit synthesis of Taxol from an endophytic fungus in host *Taxus* plants. Front. Microbiol. 4:3. 10.3389/fmicb.2013.00003 23346084 PMC3550802

[B72] Stierle A. Strobel G. Stierle D. (1993). Taxol and taxane production by *Taxomyces andreanae*, an endophytic fungus of Pacific yew. Science 260, 214–216. 10.1126/science.8097061 8097061

[B73] Tsukimoto M. Nagaoka M. Shishido Y. Fujimoto J. Nishisaka F. Matsumoto S.. (2011). Bacterial production of the tunicate-derived antitumor cyclic depsipeptide didemnin B. J. Nat. Prod. 74, 2329–2331. 10.1021/np200543z 22035372

[B74] Tsuruo T. Oh-hara T. Iida H. Tsukagoshi S. Sato Z. Matsuda I.. (1986). Rhizoxin, a macrocyclic lactone antibiotic, as a new antitumor agent against human and murine tumor cells and their vincristine-resistant sublines. Cancer Res. 46, 381–385. 3753552

[B75] Velasco A. Acebo P. Gomez A. Schleissner C. Rodriguez P. Aparicio T.. (2005). Molecular characterization of the safracin biosynthetic pathway from *Pseudomonas fluorescens* A2- 2: designing new cytoxic compounds. Mol. Microbiol. 56, 144–154. 10.1111/j.1365-2958.2004.04433.x 15773985

[B76] Wakimoto T. Egami Y. Nakashima Y. Wakimoto Y. Mori T. Awakawa T.. (2014). Calyculin biogenesis from a pyrophosphate protoxin produced by a sponge symbiont. Nat. Chem. Biol. 10, 648–655. 10.1038/nchembio.1573 24974231

[B77] Whitt J. Shipley S. M. Newman D. J. Zuck K. M. (2014). Tetramic acid analogues produced by coculture of *Saccharopolyspora erythraea* with *Fusarium pallidoroseum*. J. Nat. Prod. 77, 173–177. 10.1021/np400761g 24422636 PMC3993930

[B78] Wilson M. C. Mori T. Ruckert C. Uria A. R. Helf M. J. Takada K.. (2014). An environmental bacterial taxon with a large and distinct metabolic repertoire. Nature 506, 58–62. 10.1038/nature12959 24476823

[B79] Wings S. Müller H. Berg G. Lamshöft M. Leistner E. (2013). A study of the bacterial community in the root system of the maytansine containing plant *Putterlickia verrucosa*. Phytochemistry 91, 158–164. 10.1016/j.phytochem.2012.06.016 22795602

[B80] Wright A. E. Forleo D. A. Gunawardana G. P. Gunasekera S. P. Koehn F. E. McConnell O. J. (1990). Antitumor tetrahydroisoquinoline alkaloids from the colonial ascidian *Ecteinascidia turbinata*. J. Org. Chem. 55, 4508–4512. 10.1021/jo00302a006

[B81] Xu Y. Kersten R. D. Nam S.-J. Lu L. Al-Suwailem A. M. Zheng H.. (2012). Bacterial biosynthesis and maturation of the didemnin anti-cancer agents. J. Am. Chem. Soc. 134, 8625–8632. 10.1021/ja301735a 22458477 PMC3401512

[B82] Yamada Y. Kuzuyama T. Komatsu M. Shin-ya K. Omura S. Cane D. E.. (2015). Terpene synthases are widely distributed in bacteria. Proc. Nat. Acad. Sci. U.S.A. 112, 857–862. 10.1073/pnas.1422108112 25535391 PMC4311827

[B83] Yang Y. Zhao H. Barrero R. A. Zhang B. Sun G. Wilson I. W. (2014). Genome sequencing and analysis of the paclitaxel-producing endophytic fungus *Penicillium aurantogriseum* NRRL 62431. BMC Genomics 15:69 10.1186/1471-2164-15-69 24460898 PMC3925984

[B84] Ying T.-S. (1979). On *Dysosma* Woodson and *Sinopodophyllum*, Ying, gen. nov. of the Berberidaceae. Acta Phytotaxon. Sin. 17, 17–23.

[B85] Ying Y.-M. Shan W.-G. Zhan Z.-J. (2014). Biotransformation of huperzine a by a fungal endophyte of *Huperzia serrata* furnished sesquiterpenoid-alkaloid hybrids. J. Nat. Prod. 77, 2054–2059. 10.1021/np500412f 25222040

[B86] Yu J.-W. Floss H. G. Cragg G. M. Newman D. J. (2012). Ansamitocins (Maytansenoids), in Anticancer Agents from Natural Products, 2nd Edn, eds Cragg G. M. Kingston D. G. I. Newman D. J. (Boca Raton, FL: Taylor and Francis), 407–427.

[B87] Zaiyou J. Li M. Guifang X. Xiuren Z. (2013). Isolation of an endophytic fungus producing baccatin III from *Taxus wallichiana* var. *mairei*. J. Ind. Microbiol. Biotechnol. 40, 1297–1302. 10.1007/s10295-013-1320-4 23958913

